# Elevated C-reactive Protein and Role of Steroids in Cocaine-associated Levamisole-induced Vasculitis

**DOI:** 10.7759/cureus.7597

**Published:** 2020-04-09

**Authors:** Swetha Ann Alexander, Vinay Mathew Thomas, Jacqueline A Savage

**Affiliations:** 1 Internal Medicine, University of Connecticut, Farmington, USA; 2 Medicine, Hartford Hospital, Hartford, USA

**Keywords:** cocaine, levamisole, vasculitis, crp, steroid, cocaine levamisole-induced vascuiltis, levamisole-induced vasculitis

## Abstract

Levamisole is a common contaminant in cocaine and has led to the emergence of an entity known as levamisole-induced vasculitis (LIV). There is no consensus on the management of this condition. We describe a patient who presented with acute on chronic LIV who was treated with pulse dose steroids. We aim to discuss the diagnosis and current management options for LIV. We have compared seven case reports that have measured C-reactive protein (CRP) and compared the CRP levels, site involved, dose, and mode of steroid administration. We postulate that elevated CRP may warrant steroid therapy over conservative management and could lead to a possible decreased hospital stay.

## Introduction

Cocaine-associated levamisole-induced vasculitis (LIV) is becoming increasingly known ever since the first report in 2010 [1]. A total of 55 cases of LIV were reviewed by Pearson et al. in 2012, which described classic cutaneous lesions, neutropenia, and antineutrophilic cytoplasmic antibody (ANCA) positivity [2]. While ANCA positivity is typical, case reports suggest that serology can be negative or lacking in 10% of cases [3]. We describe a patient who presented with acute on chronic LIV who was treated with pulse dose steroids. We aim to discuss the diagnosis and current management options for LIV with the possible hypothesis that elevation of C-reactive protein (CRP) with systemic involvement or hemodynamic instability warrants steroid therapy [4-8].

## Case presentation

A 62-year-old African American male with a past medical history of prostate cancer status post radiation, diabetes, hypertension, polysubstance abuse, and subacute ischemic ulcers on his distal fingers presented with several days of fever, chills, and increasing pain in his fingers. He also endorsed complaints of nausea, vomiting, chest pain, and cough. He denied symptoms of abdominal pain, back pain, burning micturition, joint pains, other skin rashes, or ulceration.

**Figure 1:**
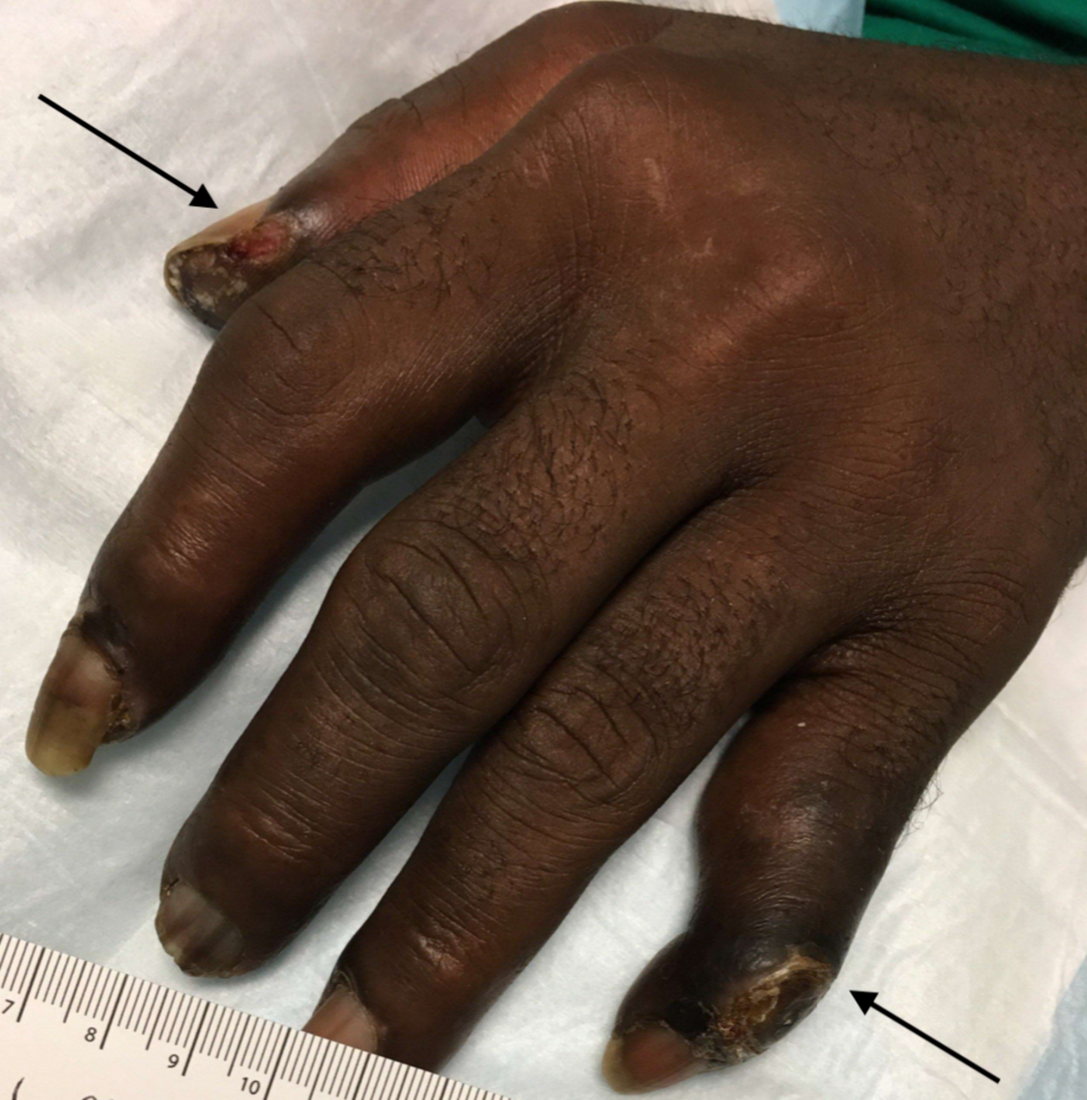
Two digits of left hand showing dry gangrene with no evidence of purulence

**Figure 2:**
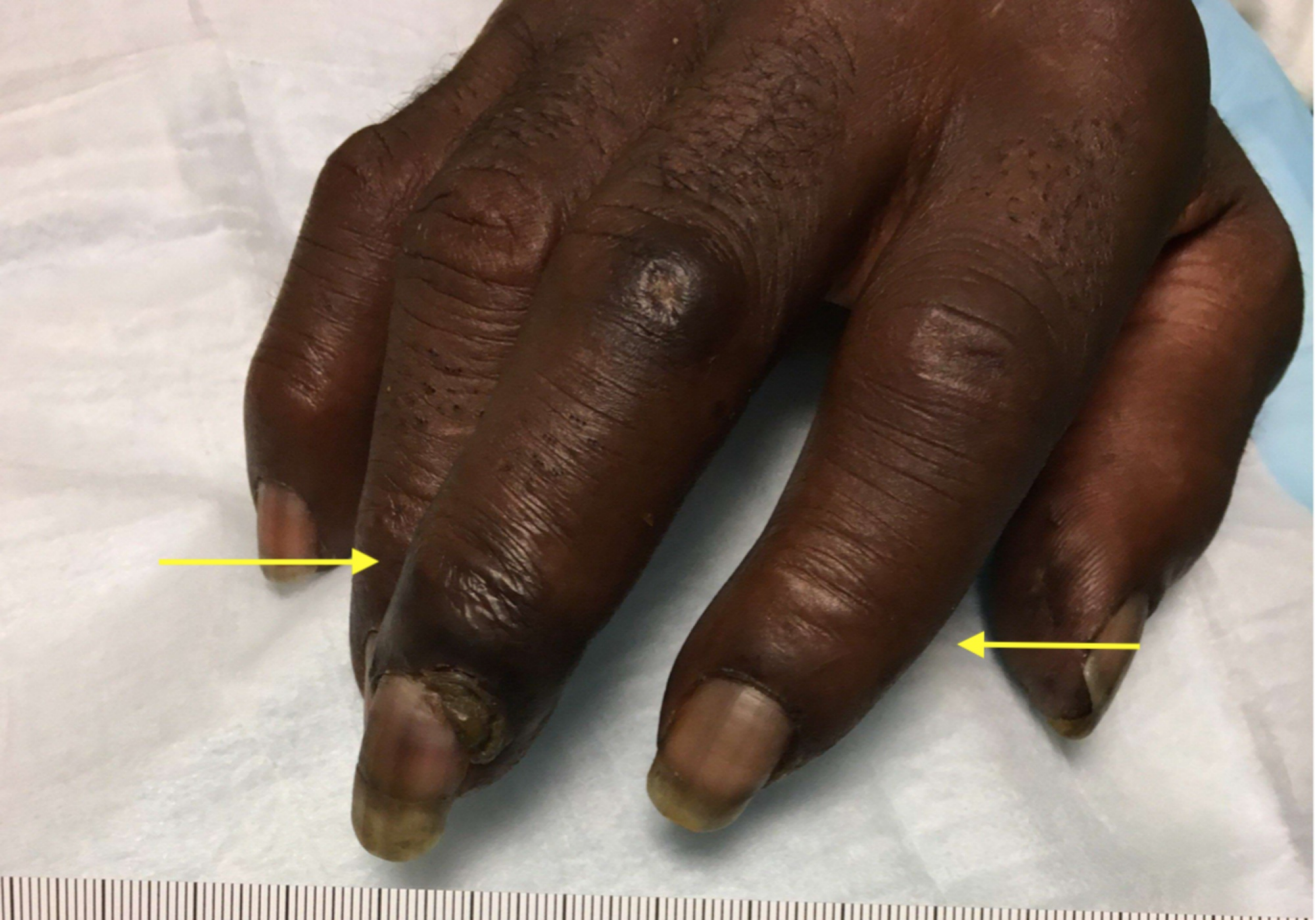
The right hand showing evidence of dry gangrene

On presentation, he was found to be febrile with a temperature of 101.2°F. He was found to be hemodynamically stable and tachycardic with a heart rate of 110 beats per minute. Physical exam was significant for well-demarcated dry gangrene on the two digits of the left hand (Figure 1). The patient was also noted to have gangrene of two digits on the right hand (Figure 2). The left hand was more painful than the right.

 

Lab values were significant for an elevated white blood cell count of 11.5/µL, lactic acid of 2.2 mmol/L, erythrocyte sedimentation rate of 126 mm/hour, and CRP of 18.6 mg/L. Urine toxicology was found to be positive for cannabinoids and cocaine. An electrocardiogram showed sinus tachycardia.

Comprehensive imaging studies including CT of chest, abdomen, pelvis, and x-ray of bilateral hands were unremarkable for an acute infectious process or pulmonary embolus. The results of all the imaging tests were normal, with no noteworthy findings. 

Given the lack of alternative data as to the etiology for his persistent intermittent fever and leukocytosis, he was started on broad-spectrum antibiotics (vancomycin and cefepime) to cover for an acute infectious process such as cellulitis in his hands.

During the hospital stay, he continued to spike intermittent fevers despite adequate antibiotic coverage (vancomycin) for possible skin and soft tissue infection of his fingers. On day 6, there was no improvement and blood cultures had no growth. Thus, investigations were done to look for alternative causes of the fever such as an autoimmune etiology. Positive results included antinuclear antibody (ANA) at 1:1,280 with a speckled pattern, rheumatoid factor at 32 IU/mL, and elevated beta-2 microglobulin. Negative results included anti-cyclic citrullinated peptide antibody, hepatitis B and C, antiphospholipid studies, cryoglobulins, and ANCA. His dilute Russell viper venom time, fibrin degradation products, serum protein electrophoresis, and quantitative immunoglobulins were normal.

Based on inconclusive test results, as stated above, the most likely diagnosis seemed to be acute on chronic cocaine-associated LIV in the setting of active cocaine use.

Other differentials were ruled out such as cryoglobulinemia-induced vasculitis, Buerger's disease (with history of active smoking), infectious etiology, such as hepatitis C and hepatitis B, antiphospholipid antibody syndrome, and disseminated intravascular coagulation.

**Figure 3:**
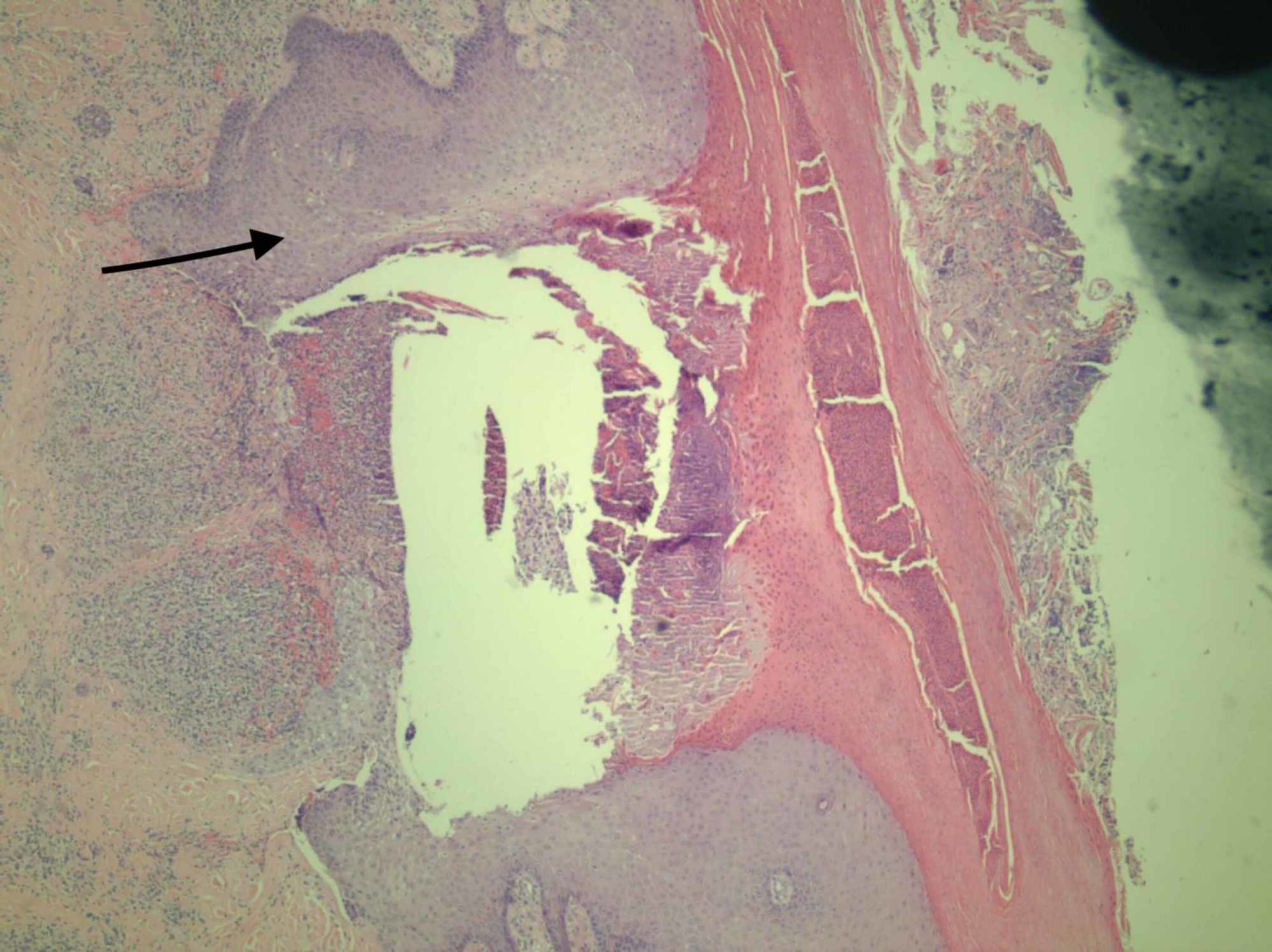
Necrotic epidermis with edema and inflammation around vessels (as depicted by arrow)

**Figure 4:**
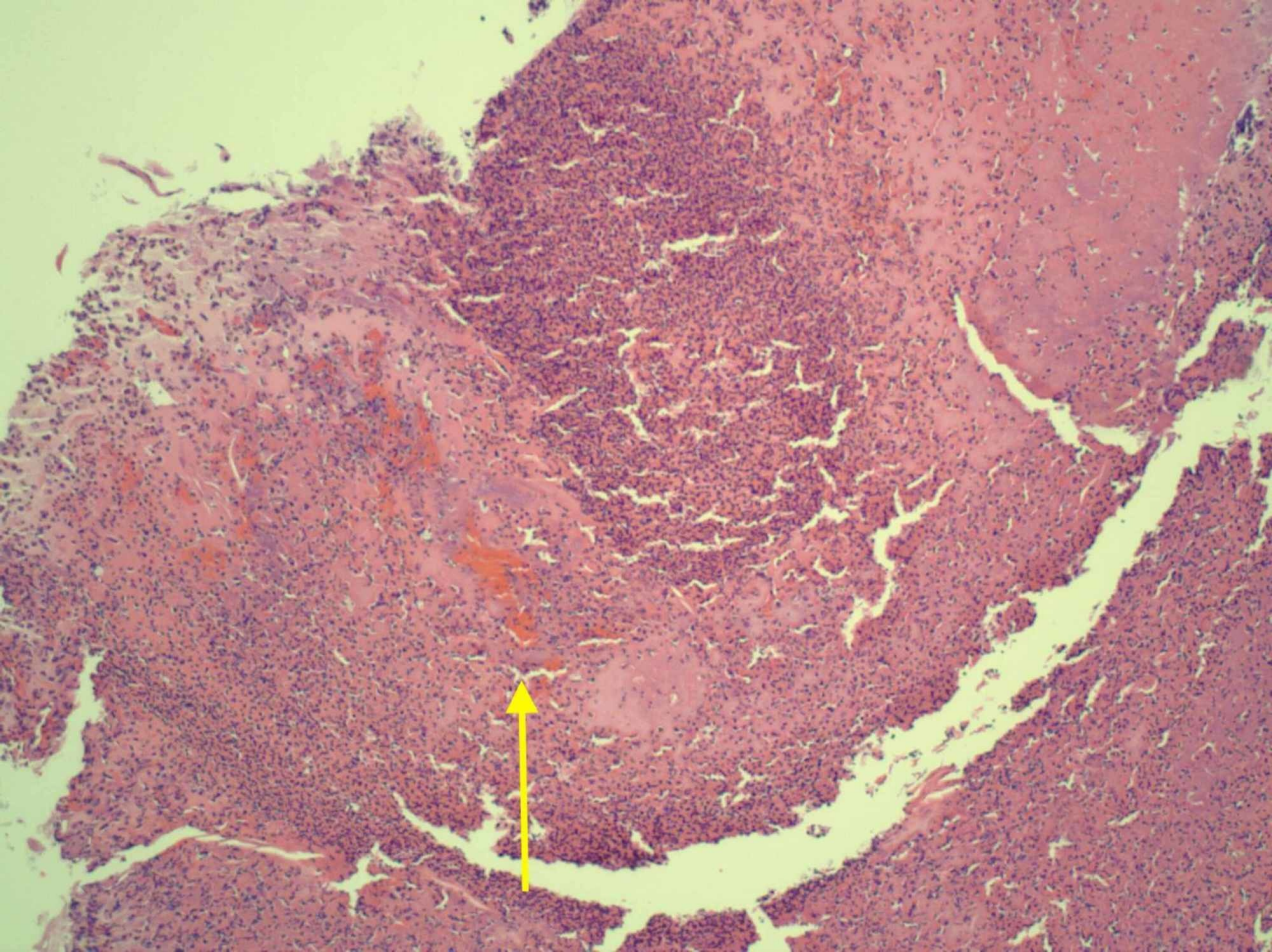
Debrided tissue showing dermal blood vessel with thrombus (yellow arrow), with wall necrosis, and a neutrophilic infiltrate

Our patient was started on pulse dose steroids at 1.5 mg/kg/day and within 24 hours, his fever resolved along with symptomatic improvement of the pain in his hands. A punch biopsy taken at the time of admission before treatment was sent to the pathology lab and was consistent with vasculitis. The punch biopsy showed edema and inflammation around the blood vessels (Figure 3). The punch biopsy also showed dermal blood vessels with thrombus formation, wall necrosis, and a neutrophilic infiltrate (Figure 4). The patient was then subsequently discharged and asked to follow up with rheumatology as an outpatient for a steroid taper. The patient was discharged on 40 mg prednisone and was asked to taper the dose by 10 mg every five days.

## Discussion

Levamisole was discovered to be a contaminant in cocaine in 2004, and the frequency rapidly increased leading to the emergence of the entity known as LIV [9]. The US Department of Justice has estimated about 70% of seized cocaine to be contaminated with levamisole in 2009 [1].

Levamisole was introduced as an antiparasitic agent with clinical benefit shown in colorectal cancer. It lost its use in clinical practice due to significant side effects such as agranulocytosis. The addition of levamisole to cocaine is presumed to lengthen the duration of cocaine’s effect as they both increase dopamine levels in the brain. It is also not detected by crude street tests for testing the purity of cocaine and will not be detected in the routine toxicological exam, due to its short half-life [9].

Levamisole influences immune function by enhancing the action of T cells and macrophages and by augmenting processes such as phagocytosis and chemotaxis. Levamisole is also thought to induce the dendritic cells towards a TH1-mediated response [10,11]. This could explain the immune-mediated vasculitis that is observed with levamisole.

The first case report proposing cocaine-associated LIV was described in 2010 after which multiple case reports have tried to describe typical clinical features and lab findings [1]. One of the largest studies reviewed 55 case reports and have tried to define a few specific clinical features such as cutaneous findings of reticulated and bullae purpuric lesion(s) with typical distribution (84%) in lower extremities while the classic site appears to be the ears (73%). Beyond cutaneous manifestations, arthralgias, hypersensitivity pneumonitis, and renal failure have also been described. The most common lab finding appears to be neutropenia which is consistent with the side effect of levamisole. The immunomodulatory action of levamisole has led to ANCA positivity, especially perinuclear ANCA has been seen in 88% of cases. ANA positivity has also been reported in around 50% of patients. Skin biopsy of the lesions has revealed evidence of vasculitis, thrombosis, or both. Treatment can be conservative as well as with steroids [2]. No benefit of steroids has been proven; however, pathophysiology of steroids being beneficial has been hypothesized to be due to its anti-inflammatory and immunosuppressive properties [1].

There is no consensus in the literature on the management of this condition. Supportive care and abstinence of cocaine use can result in the resolution of symptoms. We propose that patients with LIV presenting with elevated CRP and systemic involvement or hemodynamic instability would stand to gain from early treatment with steroids.

CRP is an acute-phase reactant that has gained much popularity for playing an active role in the process of inflammation [12]. In patients with elevated CRP, there seems to be a component of increased inflammation. When patients have systemic involvement of vasculitis or hemodynamic instability, it can be postulated that they stand to gain from the dual mechanism of immunosuppressive and anti-inflammatory action of steroid therapy.

A few cases of reports with elevated CRP who have benefited from steroid therapy are outlined in Table 1 [4-8].

**Table 1:** Comparison of CRP levels and steroid use CRP, C-reactive protein

Case	Age	Sex	CRP level (mg/L)	Site involved	The initial choice of steroid	Route of administration	Initial dose to taper	Study
1	36	F	67.7	Ears, breasts, buttocks	Methylprednisolone	Intravenous steroid	50 mg	Pavenski et al. [7]
2	41	F	50.1	Ears, cheeks, arms, thighs	Prednisone	Oral	50 mg	Pavenski et al. [7]
3	36	F	213.5	Ears, fingertips, breasts, legs	Prednisone	Oral	50 mg	Pavenski et al. [7]
4	42	F	48.5	Upper extremities, breasts	Prednisone	Oral	30 mg	Gross et al. [5]
5	49	M	11.3	Ears, legs, arms, trunk	Prednisone	Oral	40 mg	Mandrell et al. [6]
6	35	F	4.7	Lower extremities, buttocks, elbows, forearms	Prednisone	Oral	60 mg	Bhinder et al. [4]
7	45	M	Elevated	Ears, extremities	Prednisone	Oral	20 mg	Ullrich et al. [8]

Some associations that can be seen among this cohort of patients include a female predominance, as well as the use of steroid doses greater than 50 mg when larger muscle groups are involved and CRP levels are greater than 50 mg/L. There is some evidence of steroid therapy being beneficial especially with systemic involvement in addition to conservative measures [8]. In the Pearson et al study, 66% of patients found to have elevated CRP and systemic involvement showed the need for steroid treatment in addition to conservative measures [2]. Since our patient had a complicated and prolonged hospital course and did not improve despite conservative measures, we speculate if early steroid initiation could have led to better symptom management, decreased the need for extensive investigation, and resulted in decreased hospital stay. 

## Conclusions

As we reflect on the course of our patient, it can be hypothesized that immunosuppression with steroids can result in the resolution of recalcitrant cases as this counteracts the immunomodulatory effect of levamisole which induces T-cell-activated antibodies. However, further studies are needed to clarify when immunosuppression is necessary to achieve remission. Based on what we have encountered from our patient and a few other patients whose CRP has been reported, we hypothesize that CRP, which is a marker of inflammation, can help guide therapy as to whether steroid initiation would be beneficial than conservative measures alone. The requirement of higher doses of steroid therapy is seen as the CRP levels rise, which can be correlated with the hemodynamic status. We can conclude that in a hemodynamically unstable patient with elevated CRP, early steroid administration would prove to have more benefits than conservative measures alone. However, it would be prudent to rule out infectious processes first, with imaging and cultures, since CRP could be elevated in infectious processes too. 
